# Unravelling social status in the first medieval military order of the Iberian Peninsula using isotope analysis

**DOI:** 10.1038/s41598-024-61792-y

**Published:** 2024-05-14

**Authors:** Patxi Pérez-Ramallo, Carme Rissech, Lluis Lloveras, Mary Lucas, Dionisio Urbina, Catalina Urquijo, Patrick Roberts

**Affiliations:** 1 Department of Archaeology and Cultural History, University Museum, Norwegian University of Science and Technology (NTNU), Erling Skakkes Gt 47B, 7491 Trondheim, Norway; 2 isoTROPIC Research Group, Department of Archaeology,, Max Planck Institute of Geoanthropology, Kahlaische Str. 10, 07745 Jena, Germany; 3 Unitat d’Anatomia I Embriologia Humana, Dept de CiènciesMèdiques Bàsiques, Facultat de Medicina I Ciències de LaSalut, , Universitat Rovira I Virgily, 43201 Reus, Tarragona, Spain; 4 SERP, Departament d’Història I Arqueologia, Universitat de Barcelona, 08001 Barcelona, Spain; 5 Arctic University Museum of Norway, UiT-the Arctic University of Norway, 9006 Tromsø, Norway; 6 Archaeologists, Independent Researcher. ArchaeoSpain Directors. Juan Gavala 2, 16555 Carrascosa del Campo Cuenca, Spain; 7 Department of Archaeology, Max Planck Institute of Geoanthropology, Kahlaische Str. 10, 07745 Jena, Germany

**Keywords:** Mass spectrometry, Carbon cycle, Environmental social sciences

## Abstract

Medieval Iberia witnessed the complex negotiation of religious, social, and economic identities, including the formation of religious orders that played a major role in border disputes and conflicts. While archival records provide insights into the compositions of these orders, there have been few direct dietary or osteoarchaeological studies to date. Here, we analysed 25 individuals discovered at the Zorita de los Canes Castle church cemetery, Guadalajara, Spain, where members of one of the first religious orders, the Order of Calatrava knights, were buried between the 12th to 15th centuries CE. Stable carbon (δ^13^C) and nitrogen (δ^15^N) isotope analyses of bone collagen reveal dietary patterns typical of the Medieval social elite, with the Bayesian R model, ‘Simmr’ suggesting a diet rich in poultry and marine fish in this inland population. Social comparisons and statistical analyses further support the idea that the order predominantly comprised the lower nobility and urban elite in agreement with historical sources. Our study suggests that while the cemetery primarily served the order's elite, the presence of individuals with diverse dietary patterns may indicate complexities of temporal use or wider social interaction of the medieval military order.

## Introduction

The conquest of the Iberian Peninsula by the Umayyad Caliphate during the years 711 to 726 CE ushered in a novel social, economic, and political landscape^1–3^. This transformation was a significant departure from the Late Antique period, characterized by the governance of the Visigoth kingdom of Toledo, the successor to the Roman Empire, from the 5th to the early 8th centuries CE^1,4^. Subsequently, until the 15th century CE, the Iberian Peninsula bore witness to a complex interplay of coexistence and conflict between two broad sociopolitical groupings characterized by the religious affiliations of their local leaders and rulers, namely Islam and Christianity^1,5,6^. On one side stood the diverse Christian realms in the northern regions of the peninsula, who self-proclaimed a legacy from the Visigoth kingdom^1^. Conversely, in the central and southern reaches of the Iberian Peninsula, collectively known as al-Andalus, were the various political entities that succeeded the Umayyad Caliphate (711–750 CE), including the Emirate and Caliphate of Córdoba (756–1031), the Almoravid and Almohad kingdoms (1090–1220 CE), and the Taifas kingdoms (1031–1090, 1220–1492 CE)^7^. The origins and evolution of the concept of the "crusade", which emerged in Western Europe towards the close of the 11th century CE, catalysed a series of events until the 14th century CE^8^. This included the territorial expansion of the Germanic peoples into eastern Europe (the "*Drang nach Osten*"), the conquest of the Near East by Western Europe Latin forces and the noteworthy southward expansion of the northern Christian kingdoms, such as Castile, Leon, Portugal, and Aragon, into the central and southern Iberian Peninsula, and the Balearic Islands^1,4^. Concurrently, the need for a standing army to defend newly acquired territories and to protect holy sites and pilgrims, such as Jerusalem and Santiago de Compostela, stimulated the formation of diverse military orders, among them the Order of the Temple of Solomon, the Order of Saint John of Jerusalem, and the Teutonic Order^9,10^.

**Figure 1:**
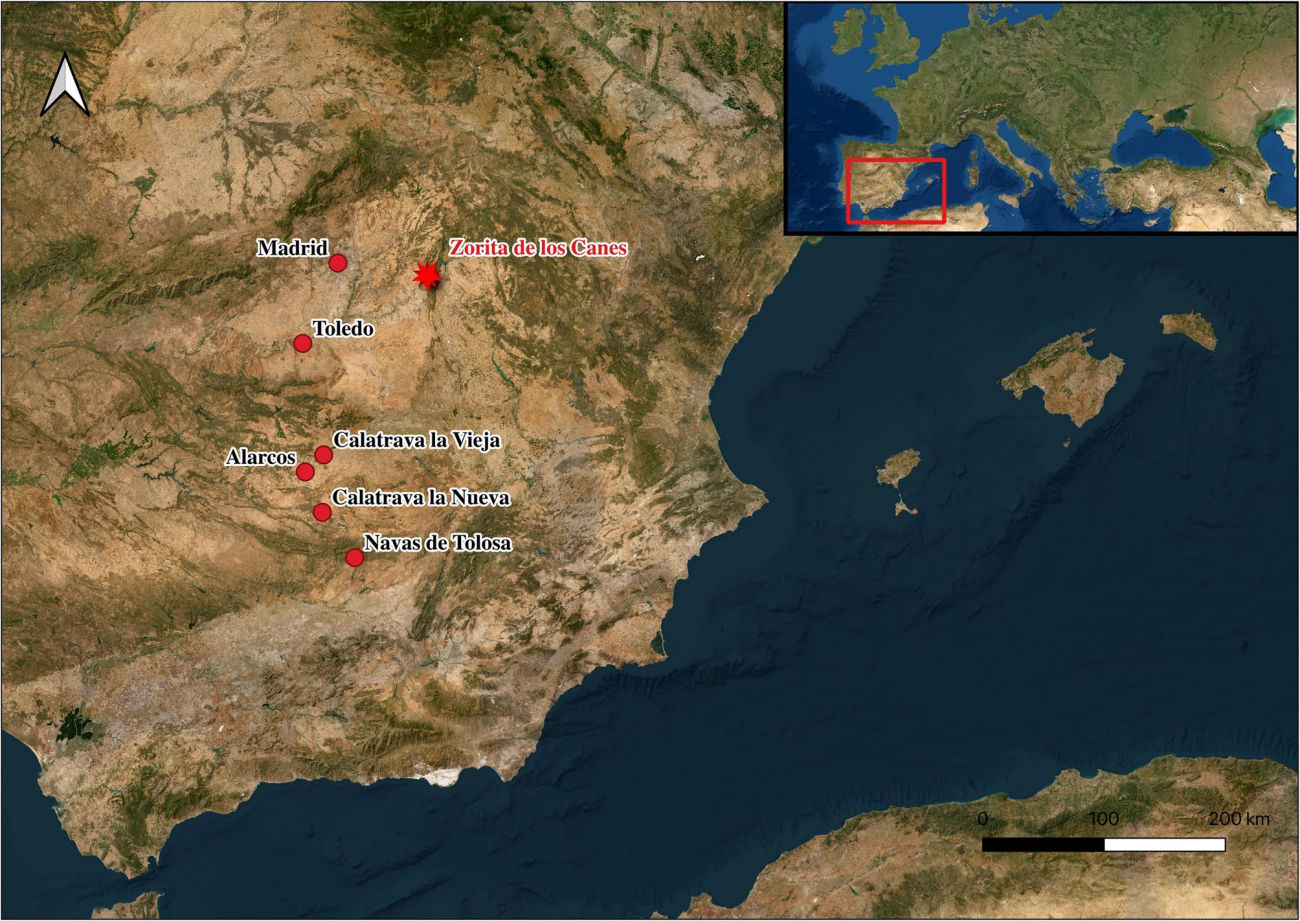
Map of the Iberian Peninsula showing the location of Zorita de los Canes and the other sites mentioned in this study. This map was generated using the open access software QSIG 3.28.1-Firence (https://qgis.org/es/site/).

Between the 11th and 13th centuries CE, the Iberian Peninsula^1^ was marked by a series of conflicts and shifts in power, involving the exchange of fortresses and intricate interactions among Christian, Muslim, and Jewish communities^2,3^. These centuries were key in shaping the Iberian Peninsula's subsequent history and the trajectory of Christian conquest. Intermittent clashes between Muslim and Christian forces gave rise to border areas characterized by warfare and political instability, even as communities interacted within urban centers^1^. This period of history in the Iberian Peninsula, which were determinative of its territorial expansion model and societal structure, culminated in the emergence of its own local military orders from the 12th century CE onward^11,12^. These orders played a pivotal role in defending borders and participating in the conquest of new territories. The first of these was the Order of Calatrava, established in the Iberian Peninsula by Abbot Raimundo de Fitero in the Kingdom of Castile in the year 1158 CE^11,12^. Its primary mission was to safeguard the city of Calatrava la Vieja (Carrión de Calatrava, Ciudad Real, Spain). The order's influence rapidly expanded with the acquisition of additional fortresses and territories, such as the castle of Zorita de los Canes (Guadalajara, Spain) in 1174 CE (Fig. 1).

Zorita de los Canes, believed to have been founded shortly after the Umayyad Caliphate's conquest of the Iberian Peninsula in 711 CE, was a particularly important fortification at the center of these changes and conflicts. Historical records provide evidence of its existence dating back to at least 812–813 CE^13,14^. Following centuries of Muslim dominance, the territorial expansion of the Christian kingdoms, especially after the conquest of Toledo in 1085 CE, prompted a southward push. Consequently, the castle of Zorita de los Canes was integrated into the network of fortresses under the Castilian monarchy's control^11,13^. King Alfonso VIII made the strategic decision to cede the fortress to the Order of Calatrava in 1174 CE, marking the onset of a period of heightened political and military significance for the stronghold^13,14^. Owing to its strategic geographical position, Zorita de los Canes held particular significance as a frontier settlement helped by the Tajo River. It witnessed years of conflict and the transfer of power between Christian and Muslim rulers (1108–1124 CE)^13,14^. The castle also became a focal point during the Christian civil war between 1158 and 1169 CE^13,14^. After the Christian defeat at the Battle of Alarcos (1195 CE), the Order of Calatrava lost its main fortress at Calatrava la Vieja (Fig. 1). This temporarily made the castle of Zorita de los Canes the main stronghold of the order^13,14^. However, in 1212 CE, under the leadership of King Alfonso VIII of Castile, a coalition of Christian peninsular kingdoms and various military orders, including the Order of Calatrava and European volunteers, achieved a key victory at Navas de Tolosa, near the town of Santa Elena, Jaén, Spain^1,11,12^. This victory also meant the recovery of the old fortress of Calatrava la Vieja (Carrión de Calatrava, Ciudad Real, Spain), and the transfer of the headquarters of the order once again, but this time to the Castle-Monastery of Calatrava la Nueva (Aldea del Rey, Ciudad Real, Spain). The territorial progress of Christian forces, which culminated in 1492 CE with the capture of the city of Granada, ensured the continued control of the castle by the warrior monks of Calatrava until its abandonment in the 16th century CE^13,14^.

The Order of Calatrava emerged in the Kingdom of Castile, situated in what would become the southernmost region of the kingdom on the border with al-Andalus. Its establishment was prompted by the need to replace the order of the Temple and address the looming threat of Almohad attacks^11,12^. The monarchy played a key role in the formation of the order, collaborating with Cistercian monks^11^. The order itself comprised both religious and lay members who shouldered military responsibilities^11^. Commencing in the 13th century CE, this militia underwent a process of aristocratization, leading to an increasing secularization. The upper echelons of the hierarchy were dominated by prominent noble families. The nobility contributed human resources, established familial agreements, and made substantial property donations. Consequently, the Order of Calatrava became an institution endowed with significant material and monetary resources^11^. They also wielded influence in the kingdom's politics as the centuries unfolded. Despite the vow of poverty taken by its members, this commitment was frequently compromised. Members perceived their affiliation not solely as a religious journey for the salvation of their souls but also as an avenue for economic betterment and social advancement. This is partly a consequence of the fact that the order primarily recruited its members from the lower nobility or urban oligarchies, constituting the majority of knights within the institution^11^. The military force of the order encompassed a spectrum ranging from Freire Knights (heavy cavalry with their own horses and entourage), Freire Sergeants (fighting on horseback but with more simple weapons and dictated by the highest lay and ecclesiastical hierarchies), Associated Fighters (temporary volunteers, primarily knights), Mercenaries, and Vassals.

Yet, despite historical insights into the social origins and compositions of the order, relatively little is known about how these distinctions were expressed in terms of dietary and economic status^9^. Medieval historical records from northern Iberia reveal distinct dietary patterns shaped by social status, historical circumstances, and geography. The diets of economic elites were marked by significant meat consumption, particularly poultry and young animals, indicating a luxury accessible only to the wealthiest^15–17^. Urban areas favored beef and lamb^18^. Fish played a crucial role for both elites and urban residents, with river fish, especially in the Kingdom of Castile, becoming a staple due to religious restrictions on meat^19^. Despite these constraints, historical sources indicate a thriving trade in marine fish to Castilian cities, emphasizing its widespread availability even inland^19^. Crop cultivation in the Middle Ages encompassed wheats, barley, millets, rye, and oat, with rye being the most cultivated cereal, while wheat was considered a luxury for the social elite^16,20,21^. Rural peasants, constituting the majority, based their diets on local cereal crops, varying by region^22^. Dairy products and meat were less accessible to rural populations, and individuals of lower social status often turned to *Panicum miliaceum* or millet during poor harvests or famine^16,18,21,23^. However, to date there have been no detailed studies of religious order members from this time period.

**Figure 2:**
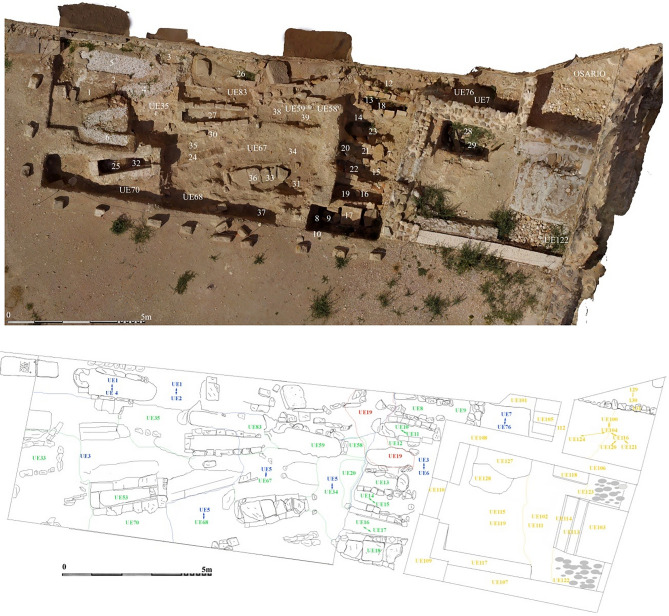
Photogrammetry and map of the medieval Christian cemetery know as Corral de los Condes or Corral of the Counts (11th–15th centuries CE) discovered and excavated in the castle of Zorita de los Canes.

Since 2014, a comprehensive series of archaeological excavations has been carried out at the Castle of Zorita de los Canes. This research endeavor has been made possible through a collaborative partnership between the Davidson Day School of North Carolina and the professional archaeological firm, ArchaeoSpain. These excavations uncovered a Christian cemetery located within the expansive esplanade of the Corral de los Condes (Fig. 2). The precise commencement of its usage as a cemetery remains a topic of debate; however, some scholars propose that it may have originated towards the conclusion of the  12th century and spanning to the 15th century CE^24^. Osteological studies have revealed that most individuals interred in this cemetery were primarily adult men of varying ages. Significantly, signs of trauma have been identified in several of these individuals, indicative of violent incidents, probably battle wounds^25^, in keeping with primary use as a cemetery for the Order of Calatrava. Nevertheless, amidst these remains, one woman and a child have been unearthed, giving rise to diverse hypotheses regarding the reasons for their burial in a location that initially appeared to be designated for members of the Order of Calatrava. These discoveries raise questions about the historical context and social composition of at least some parts of the cemetery. To illuminate the dietary practices and social stratification of those interred within the fortress, particularly individuals with affiliations to the Order of Calatrava, a comprehensive stable isotope analysis encompassing carbon (δ^13^C) and nitrogen (δ^15^N) was systematically conducted. This in-depth examination was extended to both human (n = 25) and faunal (n = 19) remains from the precincts of the Castle of Zorita de los Canes. To enhance the interpretation of our findings, we utilized a Bayesian model implemented through the R package Stable Isotope Mixing Models (SIMMr)^26^ to quantitatively assess the diets of the individuals studied at Zorita de los Canes Castle. Beyond its substantial contributions to our understanding of the dietary patterns within military orders of the past, this interdisciplinary research effort offers profound insights into the historical and archaeological import of the Castle of Zorita de los Canes.

## Results

**Figure 3:**
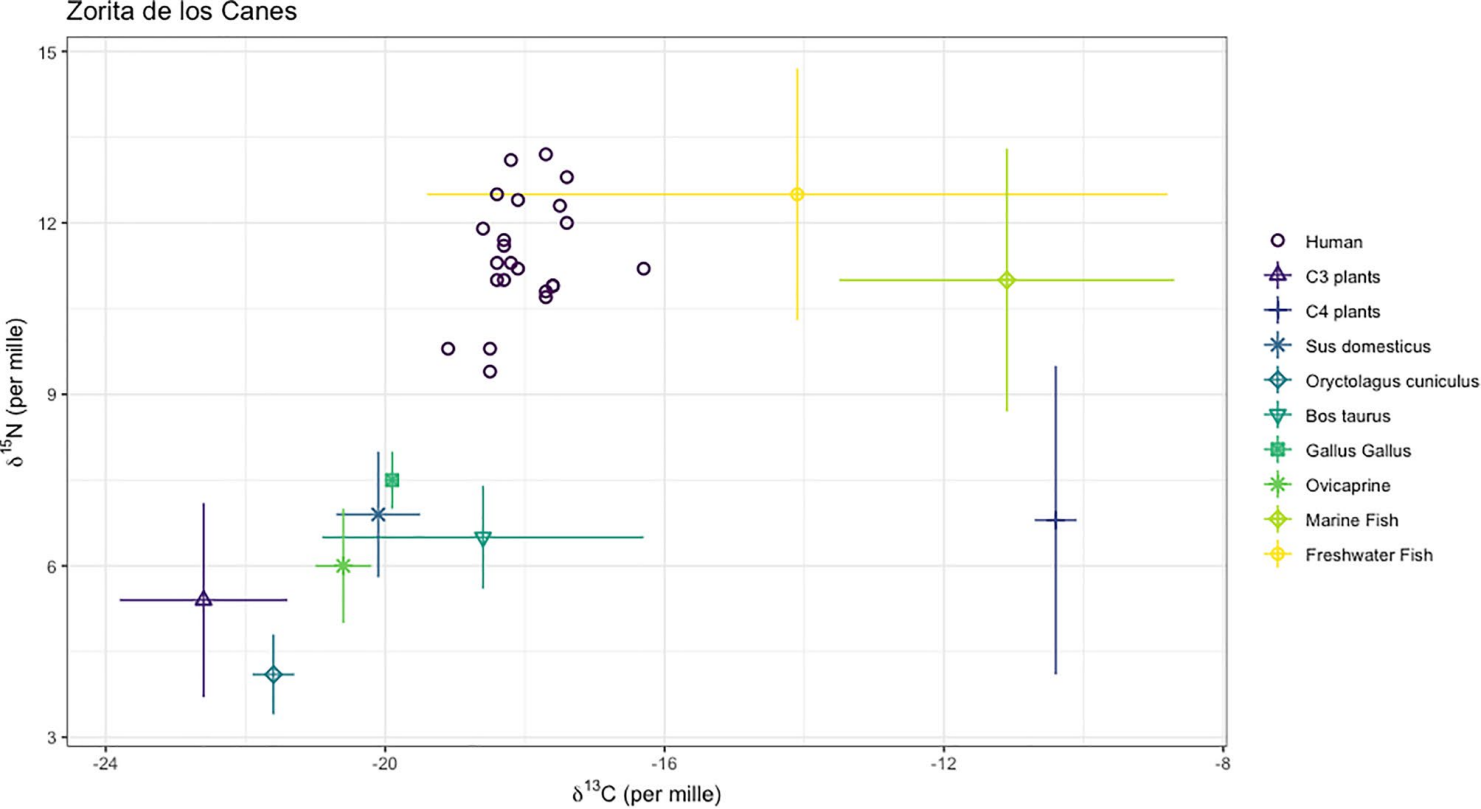
δ^13^C and δ^15^N of fauna and humans analysed in the present study and C_3_ cereals by Knipper et al. (2020)^27^, C_4_ cereals by Nitsch et al.^28^, marine fish by Alexander et al.^17^, López-Costas and Müldner^29^, and Mion et al.^30^; and freshwater fish by Mion et al.^30^.

From the 25 human and 19 fauna samples, we selected 1 osteological sample per individual for δ^15^N and δ^13^C analyses. Following, the rejection of samples that failed to meet collagen quality requirements (C:N atomic ratios, % collagen) we were left with a total of 43 from 44 samples, after rejecting the individual COC(CL)17 because of lack of collagen (Table 1). Stable isotope results for all analysed terrestrial fauna from the castle of the Zorita de los Canes (n = 19) range between 3.3 ‰ and 8.1 ‰ for δ^15^N (Mean ± SD = 5.9 ± 1.4 ‰) and between − 22.0 ‰ and − 16.1 ‰ for δ^13^C (Mean ± SD = − 20.4 ± 1.3 ‰) (Fig. 3; Table 1). A statistical comparison among terrestrial species revealed significant differences (determined using Kruskal–Wallis tests and Mann–Whitney pairwise test for equal medians) in δ^15^N and δ^13^C values. These distinctions were primarily observed between *Oryctolagus cuniculus* and *Bos taurus*, *Gallus gallus*, and ovicaprines for both δ^15^N and δ^13^C values. Additionally, significant differences were found between ovicaprines and *Gallus gallus* specifically for the δ^15^N values (see Tab. S3.1 and S3.2). However, we did not observe statistically significant differences between omnivores (*Gallus gallus* and *Sus scrofa*) (Mann–Whitney pairwise test for equal medians; p-value > 0.05) (Sup. Mat. Table S3.1.).

**Table 1:** Stable isotopes of δ^13^C and δ^15^N of human and fauna from Zorita de los Canes castle *Presence of trauma/s.

References	Humans							
	Tomb reference	Sex	Age	δ^15^N	δ^13^C	%N	%C	C/N ratio
COC(CL)01	T.1 (1)*	M	45–50	11.3	− 18.2	15.53	42.23	3.2
COC(CL)02	T.2*	M	50–55	9.8	− 19.1	15.94	43.69	3.2
COC(CL)03	T.34*	M	Ind. Adult. (> 20)	11.3	− 18.4	16.50	44.51	3.2
COC(CL)04	T.27 (3)*	M	35–45	10.8	− 17.7	15.39	42.53	3.2
COC(CL)05	T.4 (1)*	M	45–55	12.4	− 18.1	15.15	41.24	3.2
COC(CL)06	T.39*	M	Ind. Adult. (> 20)	10.9	− 17.6	10.18	27.23	3.1
COC(CL)07	T.37*	M	32–42	13.2	− 17.7	12.89	34.75	3.1
COC(CL)08	T.13*	M	50–60	11.0	− 18.3	16.06	44.26	3.2
COC(CL)09	T.18 (1)*	M	45–55	12.5	− 18.4	16.64	45.88	3.2
COC(CL)10	T.17 (1)*	M	32–37	11.7	− 18.3	15.05	40.77	3.2
COC(CL)11	T.27 (2)*	M	45–49	10.7	− 17.7	13.42	35.80	3.1
COC(CL)12	T.21*	M	31–65	11.0	− 18.4	15.36	41.87	3.2
COC(CL)13	T.12*	M	40–48	12.8	− 17.4	13.17	36.49	3.2
COC(CL)14	T.15*	M	50–60	11.2	− 18.1	16.80	45.07	3.1
COC(CL)15	T.35*	M	26–31	11.6	− 18.3	16.20	43.80	3.1
COC(CL)16	T.31*	M	28–29	12.3	− 17.5	15.40	41.36	3.1
COC(CL)17	T.4(2)*	M	45–49	-	-	-	-	-
COC(CL)18	T.17 (2)*	M	28–32	11.9	− 18.6	16.99	46.53	3.2
COC(CL)19	T.18 (2)*	M	30–40	13.1	− 18.2	16.24	45.11	3.2
COC(CL)20	T.25*	F	38–42	9.8	− 18.5	15.56	41.50	3.1
COC(CL)21	T.16*	M	19–21	11.2	− 16.3	5.48	14.21	3.0
COC(CL)22	T.30*	M	20–25	10.9	− 17.6	16.70	45.12	3.2
COC(CL)23	T.27 (1)*	M	17–18	12.0	− 17.4	14.92	39.22	3.1
COC(CL)24	T.1 (4)	Indeterminate	0.8–1.5	14.6	− 17.9	16.57	45.00	3.2
COC(CL)25	T.1 (2)	M	25–35	9.4	− 18.5	22.57	64.11	3.3

Reference	FAUNA							
	Species	δ^15^N	δ^13^C	%N	%C	C/N ratio		
COC(FAU)01	*Sus domesticus*	7.7	− 19.7	12.32	32.62	3.1		
COC(FAU)02	*Sus domesticus*	6.1	− 20.5	22.43	62.50	3.3		
COC(FAU)03	*Oryctolagus cuniculus*	3.7	− 21.3	14.99	40.73	3.2		
COC(FAU)04	*Oryctolagus cuniculus*	4.3	− 21.7	16.62	44.96	3.2		
COC(FAU)05	*Oryctolagus cuniculus*	3.3	− 21.2	16.09	44.11	3.2		
COC(FAU)06	*Oryctolagus cuniculus*	4.1	− 22.0	15.88	43.78	3.2		
COC(FAU)07	*Oryctolagus cuniculus*	5.2	− 21.7	14.88	41.06	3.2		
COC(FAU)08	*Bos taurus*	7.5	− 16.1	16.43	44.97	3.2		
COC(FAU)09	*Bos taurus*	5.9	− 20.5	14.28	38.40	3.1		
COC(FAU)10	*Bos taurus*	6.1	− 19.3	16.54	46.50	3.3		
COC(FAU)11	*Gallus gallus*	7.1	− 20.0	16.16	43.36	3.1		
COC(FAU)12	*Gallus gallus*	8.1	− 19.7	15.78	43.80	3.2		
COC(FAU)13	*Gallus gallus*	7.2	− 19.9	15.77	45.08	3.3		
COC(FAU)14	Ovicaprine	6.5	− 20.0	15.70	43.52	3.2		
COC(FAU)15	Ovicaprine	7.8	− 20.3	15.37	42.40	3.2		
COC(FAU)16	Ovicaprine	5.4	− 21.3	13.86	38.24	3.2		
COC(FAU)17	Ovicaprine	5.5	− 20.8	15.67	42.87	3.2		
COC(FAU)18	Ovicaprine	5.6	− 20.5	16.15	43.11	3.1		
COC(FAU)19	Ovicaprine	5.4	− 20.5	14.28	38.40	3.1

**Figure 4:**
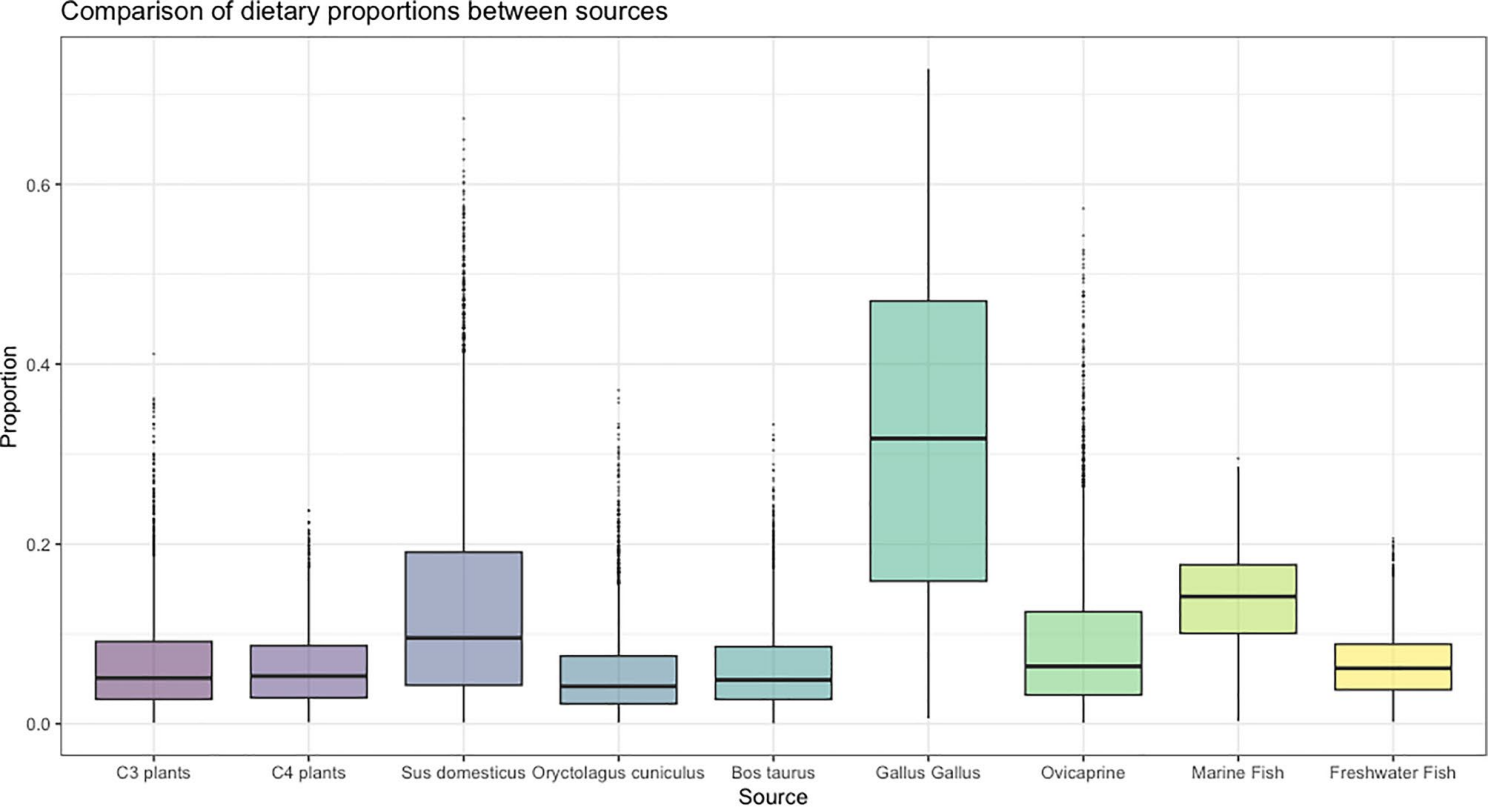
Comparison of dietary proportions between sources from the Bayesian R model ‘Simmr’.

**Figure 5:**
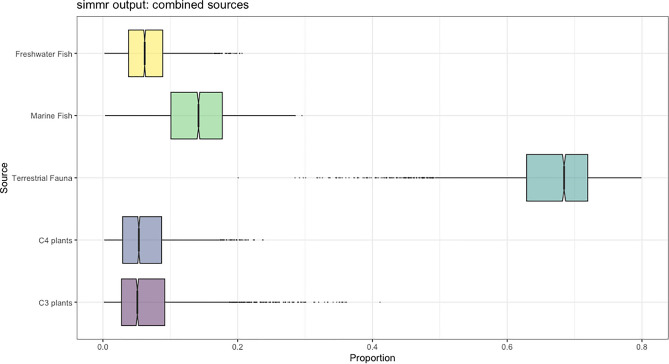
Comparison of dietary proportions between sources combining terrestrial fauna (*Sus domesticus*, *Oryctolagus cuniculus*, *Bos taurus*, *Gallus Gallus*, and Ovicaprine) from the Bayesian R model ‘Simmr’.

The δ^15^N and δ^13^C values of the human individuals analysed (n = 24) range from 9.4 ‰ to 14.6 ‰ for δ^15^N (Mean ± SD = 11.6 ± 1.2‰) and from − 19.1 ‰ to − 16.3 ‰ for δ^13^C (Mean ± SD = − 18.0 ± 0.6 ‰) (Fig. 3, Table 1). Among the adult individuals analysed here (> 20 years) (n = 23), δ^15^N values range from 9.4 ‰ to 13.2 ‰ (Mean ± SD = 11.4 ± 1.0 ‰) and δ^13^C values range from − 19.1 ‰ to − 16.3 ‰ (Mean ± SD = − 18.0 ± 0.6 ‰) (Table 1). The utilization of the Bayesian R package Stable Isotope Mixing Models (SIMMr v0.4.5)^26^ for the quantitative evaluation of the diets of scrutinized individuals at Zorita de los Canes Castle contributions (Figs. 4 and 5, Sup. Mat. S2) suggests a predominant reliance on animal protein in their diet, followed by marine fish (Mean and SD = 13.8 ± 5.3%), C_3_ plants (Mean and SD = 6.8 ± 5.7%), freshwater fish (Mean and SD = 6.6 ± 3.6%), and, lastly, C_4_ plants (Mean and SD = 6.3 ± 4.3%). Based on our input baselines, the terrestrial animals most frequently consumed were *Gallus gallus* (Mean and SD = 31.6 ± 18.2%), followed by *Sus domesticus* (Mean and SD = 13.8 ± 12.6%), ovicaprines (Mean and SD = 9.2 ± 8.5%), *Bos taurus* (Mean and SD = 6.3 ± 4.8%) and, lastly, *Oryctolagus cuniculus* (Mean and SD = 5.7 ± 5.0%) (Fig. 3, Tab. S2.4).

## Discussion

Despite the intensity of the use of a small area of land as a cemetery in the castle of Zorita de los Canes for over 300 years, disentangling the stratigraphic complexity and chronology is challenging, hindering an exact correlation of tomb typologies and their age (Fig. 2)^24^. Nevertheless, the presence of knights from the Order of Calatrava in the Corral de los Condes cemetery is evident. Historical sources, the dominance of male skeletons with evidence of abundant battel injuries, and dietary evidence of high social status all indicates that it was dominated by monk warriors or knights of the Order of Calatrava. Approximately one-third of the tombs were unaltered, providing a relative chronology through their typology, stratigraphic level, proximity to the church entrance, and the presence of a high number of penetrating injuries and blunt force trauma. Individuals predating the establishment of the Order of Calatrava in the Castle of Zorita de los Canes are most likely located in the deepest, as-yet-unexcavated, parts of this necropolis or in altered tombs with multiple individuals (e.g., skeletal elements not related to complete individuals discovered in tombs 1, 4, 17, 18, and 27). Additionally, early remains marked with order crosses were discovered, reminiscent of those found in Évora, Portugal, later recognized as the Order of Évora^31^. Most of the individuals display a significant number of penetrating stab wounds and blunt force injuries (Table 1), suggestive of violent episodes such as those in Évora^31^, may be associated with war events like the battles of Alarcos (1195 CE) or Navas de Tolosa (1212 CE). Between these two conflicts, Zorita de los Canes assumed a pivotal role as the primary fortress for the Order of Calatrava^13^, with the burial of its main members taking probably place in this fortress between 1195 and 1212 CE, until the order relocated the headquarters to Calatrava la Nueva^13,14^ (Fig. 1). The presence of a woman (n = 1) and an infant (n = 1) among the monk warriors, as well as in Évora, may be associated with the site's repopulation. However, as in Évora, determining the exact timing of their arrival and social status remains challenging^31^, and they may indicate that some individuals do come from different periods of site use. Some altered tombs with remains from more than one individual may belong to earlier Christian inhabitants, such as those involved in the civil war between the Lara and Castro families (1158 and 1169 CE)^14^. To determine the age and origin of each individual precisely, a comprehensive study involving and exhaustive radiocarbon dating will be essential. However, overall, it is clear that the majority of individuals sampled here are likely members of the standing army of the Order of Calatrava.

When comparing values from adult individuals at Zorita de los Canes (δ^15^N Mean ± SD = 11.4 ± 1.0 ‰, and δ^13^C Mean ± SD = − 18.0 ± 0.6 ‰) with those derived from Pérez Ramallo et al.^4^ research on social status differences in the medieval Iberian Peninsula, it suggests that the individuals under study belonged to the social elite of their time (see Table 2). Pérez-Ramallo et al.^4^ reconstructed individuals' diets through stable carbon and nitrogen isotope analysis, revealing significant variations in animal protein consumption when social origins were taken into account. The research of Pérez-Ramallo and colleagues encompassed various social groups, including royalty, bishops, residents of emerging northern Iberian medieval cities, and rural populations. The authors observed that individuals belonging to the Christian social elite of the time (10th–12th centuries CE) exhibited higher stable nitrogen isotope values (δ^15^N Mean ± SD = 11.8 ± 1.8 ‰, and δ^13^C Mean ± SD = − 18.6 ± 0.4 ‰) compared to urban (11th–13th centuries CE) (δ^15^N Mean ± SD = 10.5 ± 1.0 ‰, and δ^13^C Mean ± SD = − 18.7 ± 1.2 ‰) and rural populations (9th–11th centuries CE) (δ^15^N Mean ± SD = 9.5 ± 0.7 ‰, and δ^13^C Mean ± SD = − 18.5 ± 0.6 ‰). Based on historical information, we know that the social composition of the Order of Calatrava members from the 12th to the 15th centuries CE, predominantly featured individuals from the lower nobility and urban oligarchies. δ^15^N and δ^13^C values for the individuals analyzed here are overall consistent with those of a high social status. However, the presence of some individuals with lower than average nitrogen isotope values (COC(CL)02, COC(CL)04, COC(CL)06, COC(CL)11, and COC(CL)20) may also suggests members from urban (COC(CL)04: δ^15^N = 10.8 ‰ and, δ^13^C = − 17.7 ‰; COC(CL)06: δ^15^N = 10.9 ‰, and δ^13^C = − 17.6 ‰; COC(CL)11: δ^15^N = 10.7 ‰, and δ^13^C = − 17.7 ‰) and rural populations (COC(CL)02: δ^15^N = 9.8 ‰, and δ^13^C = − 19.1 ‰; COC(CL)20: δ^15^N = 9.8 ‰, and δ^13^C = − 18.5 ‰). This might highlight the diverse social origins of the order’s armed forces which, alongside the knights and sergeants, included temporary warriors, mercenaries, and vassals from the order's jurisdictional lands^11^.

**Table 2:** δ^15^N and δ^13^C measurements for medieval ‘Christians’ and ‘Muslim’ individuals available in the literature from the Iberia Peninsula excluding those potentially influenced by breastfeeding (mean, SD, and number of samples).

Site	Religion	n	δ^13^C (VPDB)	SD	δ^15^N (AIR)	SD	References
Zorita de los Canes, Guadalajara	Christian	24	− 18.0	0.6	11.4	1.0	Present Study
Medieval social elite, north-eastern Iberian Peninsula	Christian	6	− 18.6	0.4	11.8	1.8	^4^
Urban, north-eastern Iberian Peninsula	Christian	14	− 18.7	1.2	10.5	1.0	^4^
Rural, north-eastern Iberian Peninsula	Christian	20	− 18.5	0.6	9.5	0.7	^4^
Castile Royal Family, Seville	Christian	4	− 18.7	0.4	12.7	0.9	^15^
Évora, Portugal	Christian	7	− 17.6	0.5	11.3	0.3	^31^
Gandía, Valencia	Christian	24	− 17.2	1.0	10.2	0.8	^17^
Valencia	Christian	19	− 18.4	0.6	10.9	1.9	^45^
San Baudelio de Berlanga, Soria	Christian	20	− 18.3	0.5	10.3	0.5	^33^
Palacios de la Sierra, Burgos	Christian	5	− 18.9	0.8	9.4	1.5	^15^
Benipeixcar, Valencia	Muslims	20	− 16.4	0.9	10.7	0.6	^46^
Tossal de las Basses, Alicante	Muslims	14	− 18.3	0.3	11.4	0.9	^47^
El Raval, Crevillent, Alicante	Muslims	35	− 16.4	0.6	12.1	0.3	^48^
Écija, Sevilla	Muslims	39	− 19.0	0.3	10.1	1.1	^40^
La Torrecilla, Granada	Muslims	74	− 15.8	1.3	10.0	0.6	^49^
Valencia	Muslims	38	− 17.7	1.3	11.5	1.4	^46^

Regarding the only female individual in our study (COC(CL)20: δ^15^N = 9.8 ‰, and δ^13^C = − 18.5 ‰), this may provide lower values due to sex differences, which have been documented in other parts of the Iberian Peninsula^17,32,33^. However, a significant number of studies also observed no differences^4,29,34–36^. Still, the fact that we could only analyse one female individual inhibits any comprehensive comparison based on sex. Alternatively, as seen in Évora^31^, this woman, together with other male individuals that illustrated lower δ^15^N values (COC(CL)02 and COC(CL)25) (see Table 1), may be individuals attracted by the repopulation efforts in recently conquered areas. Therefore, the presence of women or individuals from other social classes among the knights of the Order of Calatrava could indicate their roles as servants or workers in the castle. On the other hand, the infant's values (COC(CL)T.1(4): δ^15^N = 14.6 ‰, and δ^13^C = − 17.9 ‰; estimated age between 0.8 and 1.5 years) clearly shows the effects of breastfeeding when compared to the adult individuals in the study. This is consistent with the expected practice of breastfeeding up to the age of 3 years for boys and up to the age of 2 years for girls during this period^37^. When comparing the values of COC(CL)T.1(4) with those of the woman analysed in this study (COC(CL)20), it was observed that the infant δ^15^N values are higher and outside the expected increment range (2–3 ‰)^38,39^. Consequently, the δ^15^N values of the mother or the person in charge of breastfeeding the infant, as sometimes another woman was substituted^37^, should range between 12.6 ‰ and 11.6 ‰. This suggests the presence of at least one more woman with a diet richer in animal and/or marine protein and, consequently, potential social status differences also among local women. However, our interpretation remains speculative until more female individuals are discovered and analysed at Zorita de los Canes, including through other techniques to examine kinship relationships (e.g., aDNA).

Our Bayesian model attempts to provide more detailed insights into the dietary sources of individuals in Zorita de los Canes (Sup. Mat. S1 and S2). Following its results, the main food consumed appears to have been *Gallus gallus* (Fig. 4), aligning with historical records that highlight poultry as a favourite among the Medieval social elite in Iberia^15–17^. This would correspond well with the social status of most Calatrava knights. The significant consumption of freshwater, and particularly marine fish, is also notable, potentially influenced by the order's affiliation with the Cistercian Order, which promoted strict adherence to religious restrictions on meat consumption^19^. Our Bayesian model indicates a notable disparity in fish consumption, with marine fish accounting for 13.5%, compared to river fish at 6.8% (Sup. Mat. S2). Given the abundance of fish in the nearby Tagus River, an inland water source, it is notable that the majority of consumed fish comes from coastal regions. This observation may further underscore the economic prosperity and connectivity of the population, enabling extensive consumption of marine-origin fish even in inland areas. The Simmr Bayesian model further highlights a preference for C_3_ plants over C_4_ plants, supporting the historical sources that indicate the social elite's favouritism toward barley or wheat rather than millet^16,20,21^. The presence of C_4_ plants may be linked to direct consumption or indirect exposure through the ingestion of animals fed with millet as fodder^21^. It might also be influenced by the site's proximity to al-Andalus, providing individuals in Zorita de los Canes access to plants commonly found in the Islamic region of the Iberian Peninsula, such as sorghum or sugar cane^16,40^. However, differences in time and location can cause significant variations in the isotopic values of plants and animals due to factors such as climate, farming practices, manuring, proximity to the coast, latitude, and altitude^41–43^. Despite our meticulous efforts to select C_3_ and C_4_ plants, as well as marine and freshwater fish from geographically closer and climatically similar areas for our Bayesian model (see Sup. Mat. Tab. S2.1), it is important to acknowledge that the variability inherent in their location and potential baseline differences through time significantly impacts the accuracy of our approach. This is evident from the wide range of deviation in the estimates produced (see Sup. Mat. Fig. S2.1; Tab. S2.3 and S2.4). Furthermore, we have included a substantial number of food sources (n = 9), which further complicates the precision of our Bayesian model. Therefore, we should approach this interpretation with caution until further analysis of local plants and freshwater fish can provide a more reliable model. In addition, the impact of an individual’s geographical origin on these dietary patterns remains uncertain and necessitates further analyses, including studies using ^87^Sr/^86^Sr, δ^18^O, and δ^34^S proxies^34,35,44^.

**Figure 6:**
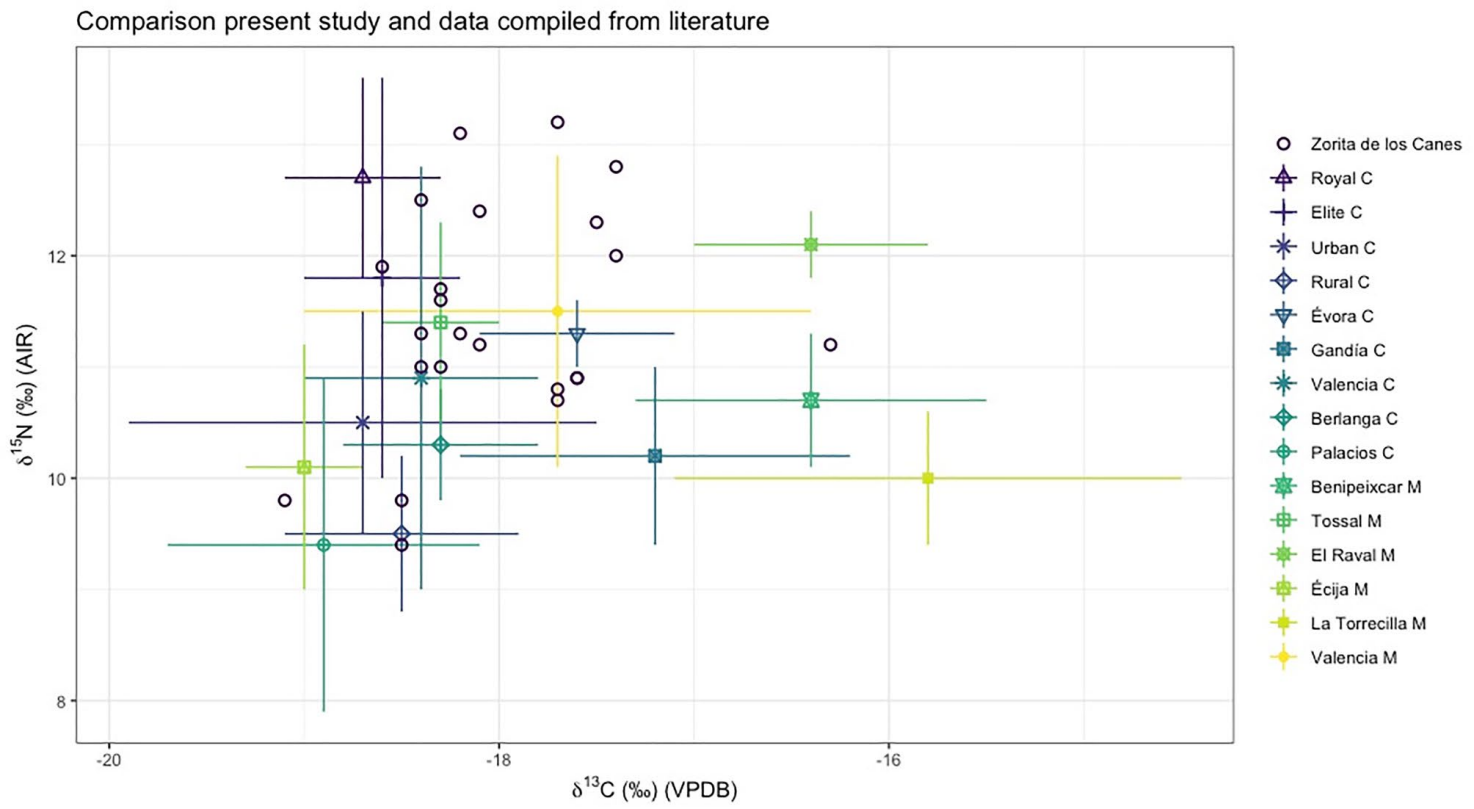
δ^13^C and δ^15^N human individuals from the present study and the ‘Christian’ and ‘Muslim’ compiled literature data (see Table 2). C = Christian; M = Muslim.

A comprehensive analysis of our findings in comparison with stable isotope analyses conducted at contemporaneous archaeological sites near Zorita de los Canes Castle provides a more nuanced understanding of socially-related dietary practices in Medieval Iberia (Table 2, Fig. 6). Our initial comparison involves knights of the Order of Évora (Portugal), fellow members of the Order of Calatrava^31^, the social elite of the Kingdom of Aragon (including two bishops, a princess, a count, and two monks)^4^, and members of the Castilian royalty buried in the Seville Cathedral^15^. Notably, δ^15^N and δ^13^C values obtained from the samples analyzed here closely resemble those in Évora. However, in contrast to those observed by Jiménez-Brobeil et al.^15^ for the Castilian Royal members buried in Seville, the individuals studied here exhibit generally lower δ^15^N values. This suggests that individuals in Zorita de los Canes belong to a social elite, yet their values are lower than those of the royalty. This aligns with historical sources indicating that the order primarily comprised the lower nobility and urban elite^11^. Nevertheless, it is noteworthy that two individuals (COC(CL)07 = δ^15^N 13.2 ‰, and δ^13^C-17.7 ‰; COC(CL)09 = δ^15^N 12.5 ‰, and δ^13^C-18.4 ‰) could be associated more closely with the higher nobility based on their nitrogen and carbon isotope values. Intriguingly, both individuals displayed perimortem trauma from a violent episode (Table 1)^25^.

A statistical comparison reveals a significant difference in the δ^13^C values of adult individuals from Zorita de los Canes compared to those of the social elite in the Kingdom of Aragon and the royalty members of Castile (Kruskal–Wallis test, and Mann–Whitney pairwise test for equal medians; p < 0.05). This distinction may be attributed to a stronger adherence to religious norms, leading to a shift from meat to fish consumption among the order members. However, it also raises the possibility of a higher intake of C_4_ plants in contrast to identified members of royalty. Additionally, comparing our results with data available in the wider literature on medieval Muslim and Christian populations geographically and chronologically close further supports our interpretation of individuals belonging to the social elite, especially when contrasting rural and urban populations (Fig. 6, Table 2). While fish consumption is evident in Zorita de los Canes, the prominence of animal protein in the individuals analyzed stands out compared to coastal populations (e.g., Benipeicar, Tossal de las Bases or El Raval) (Table 2). Caution is warranted when drawing parallels with the Muslim population of Valencia (11th–13th centuries CE), however, given factors like the potential presence of non-local individuals noted by the authors of that study^17^, as well as significant climatic and baseline variation between the regions.

Overall, our stable isotope results suggest that the Corral de los Condes cemetery from Zorita de los Canes Castle was primarily intended for knights and sergeants of the order, positions held by the high nobility (hierarchy), but particularly by the lower nobility and the urban elite. However, individuals with diets more typical of other social statuses imply that the cemetery might not have been exclusively reserved for the order's elite but also included members of lower statuses within it. Considering the order's role as a mechanism for social advancement, these male individuals may have been from the lower nobility or the urban elite with fewer material means. Future analyses of different bone or dental remains from the same individual, could reveal if there were dietary differences throughout their lives, shedding light on whether membership in the Calatrava order improved living conditions. Nonetheless, as mentioned earlier, we cannot rule out the possibility that some individuals are representative of Christian communities predating the fortress's cession to the Calatrava order in 1174 CE, or indicative of wider social repopulation of the site during times of peace. In addition, our Bayesian model is limited by the absence of local values for plants, cereals, and freshwater fish. These limitations and its high deviation and error margins must be considered when interpreting the results.

## Materials and methods

We present a comprehensive, novel isotopic dietary analysis of 25 individuals (23 adult males, 1 adult female, and 1 indeterminate child) whose skeletal remains were exhumed from the Corral de los Condes cemetery located within the historically significant Castle of Zorita de los Canes, Guadalajara, Spain (Fig. 1). Our approach involved the sampling of a rib fragment from each individual, reflecting the last 5–10 years of life due to their quick turnover^50^. To further enhance the accuracy and contextual interpretation of the isotopic data derived from human collagen, we extended our investigations to encompass 19 sets of remains (cattle, ovicaprid, pigs, and poultry), from between the 12th and the 16th centuries CE, comprising both domestic and wild fauna, also discovered within the wider confines of the castle. All data generated or analysed during this study are included in this published article, and its supplementary information files. All methods employed in this study were meticulously executed in strict adherence to relevant guidelines and regulations by the Patrimonio Cultural de Castilla la Mancha. The experimental protocols underwent rigorous scrutiny and received approval from the Zorita de los Canes town council, and those legally responsible for Cultural Heritage of Castilla la Mancha (legal representatives of the Cultural Heritage of the Government of Spain), ensuring the highest standards of ethical conduct. Additionally, it is imperative to confirm that informed consent was obtained from their legal guardians, Dionisio Urbina and Catalina Urquijo, also co-authors of this manuscript and directors of the archaeological excavation at Zorita de los Canes, emphasizing the commitment to ethical practices in this research endeavour.

### Stable isotope analysis

The dietary patterns of ancient humans can furnish insights into their social standing and origins^4,34,35^. The variability in stable carbon isotopes (δ^13^C) within terrestrial ecosystems is primarily shaped by the photosynthetic pathways employed by plants forming the foundation of the food chain^51^. Consequently, distinct and non-overlapping δ^13^C values emerge, with C_3_ plants (such as trees, shrubs, temperate grasses, and crops like wheat) exhibiting a range of approximately − 24 ‰ to − 36 ‰ (with a global average of − 26.5 ‰)^52^. By contrast, C_4_ plants (including tropical grasses and crops like maize, millet, and sugar cane) showcase a range of about − 9 ‰ to − 17 ‰ (with a global average of − 12‰)^52,53^. CAM plants (such as succulents) display δ^13^C values that overlap and fall between those of C_3_ and C_4_ plants. Due to different sources of CO_2_ in marine ecosystems, marine plants exhibit δ^13^C values more akin to C_4_ plants. These δ^13^C distinctions are transmitted up the food chain to the tissues of consumers, with an enrichment of 0.5–2 ‰ in δ^13^C observed between trophic levels^52,53^. Stable nitrogen isotope (δ^15^N) values for both terrestrial and aquatic animals vary in accordance with trophic level, escalating by + 3–6 ‰ with each successive trophic level^54^. Longer food chains and variations in nitrogen sources contribute to, on average, higher δ^15^N values among marine and freshwater consumers, although the δ^13^C values of freshwater ecosystems exhibit greater variability^55,56^.

We prepared bone samples (rib fragments) of approximately 1 g weight by breaking them into smaller fragments and removing adhering soil through abrasion using a sandblaster. These samples underwent demineralization by immersing them in 0.5 M HCl for a period of 1–7 days. After complete demineralization, the samples were rinsed three times with ultra-pure H_2_O. The residue was then gelatinized in pH3 HCl at 70 °C for 48 h. The resulting soluble collagen solution was Ezee-filtered to eliminate insoluble residues, following the method outlined by Brock et al.^57^. Subsequently, the samples were lyophilized using a freeze dryer for 48 h. In cases where we had sufficient material, approximately 1.0 mg of the resulting purified collagen was weighed in duplicate and placed into tin capsules for further analysis. The δ^13^C and δ^15^N ratios of the bone collagen were analyzed using a Thermo Scientific Flash 2000 Elemental Analyser coupled to a Thermo Delta V Advantage mass spectrometer at the Isotope Laboratory, MPI-GEA (formerly MPI-SHH), Jena. Isotopic values are presented as the ratio of the heavier isotope to the lighter isotope (^13^C/^12^C or ^15^N/^14^N) as δ values in parts per mil (‰) relative to international standards: VPDB for δ^13^C and atmospheric N2 (AIR) for δ^15^N. The results were calibrated against international standards (IAEA-CH-6 Sucrose, IAEA-N-2 Ammonium Sulfate, and USGS40 L-Glutamic Acid). Specifically, USGS40 values were ^13^C_raw_ = − 26.4 ± 0.1, ^13^C_true_ = − 26.4 ± 0.0, ^15^N_raw_ = − 4.4 ± 0.1, and ^15^N_true_ = − 4.5 ± 0.2; IAEA N-2 values were ^15^N_raw_ = 20.2 ± 0.1, and ^15^N_true_ = 20.3 ± 0.2; IAEA C6 values were ^13^C_raw_ = − 10.9 ± 0.1, and ^13^C_true_ = − 10.8 ± 0.0.

Replicated analyses of standards indicate a machine measurement error of approximately ± 0.1 ‰ for δ^13^C and ± 0.1‰ for δ^15^N. We assessed the overall measurement precision by conducting repeat extractions from a fish gelatin standard (n = 20), resulting in a precision of ± 0.1‰ for δ^13^C and ± 0.1 ‰ for δ^15^N. To ascertain purity and collagen preservation, we examined the carbon-to-nitrogen stable isotope ratio, aiming for a range of 2.9–3.6, a value typically found in fresh bone collagen^58^. The elemental mass percentages are approximately 34.8 ± 8.8 % for carbon and between 11 and 15% for nitrogen^59^. External factors, such as humic acids or salts, can alter these percentages^59^. Collagen yield, representing the percentage of collagen extracted from the bone, serves as an indicator of bone preservation quality. Fresh bone typically contains about 20% collagen. Diagenesis can cause collagen loss in the bone, to the extent that the isotopic signature obtained from a low-yield sample may no longer reflect its original isotopic signature. While sample filtration helps eliminate residues, it can also lead to a substantial loss of yield (around 40% to 60%)^60^. Ambrose and Norr^53^ set the limit at 1.2%. However, van Klinken^59^ established a minimum range between 0.5–1.0% for archaeological bones.

### Statistical analysis and the Bayesian model

Kruskal–Wallis tests and Mann–Whitney pairwise test for equal medians were applied for fauna δ^13^C and δ^15^N comparison based on their specie. The same test was applied to compare the individuals analyzed from Zorita de los Canes with those of the Castilian royal members buried at the Cathedral of Seville^15^, and the social elite individuals from the Kingdom of Aragon^4^. We employed a 5% significance level (α). Results are in Supplementary Material S3. The free software ‘PAST’ was used for all statistical analyses^61^.

We employed the Bayesian R (v4.3.1) package Stable Isotope Mixing Models (SIMMr v0.4.5) with the Markov chain Monte Carlo (MCMC) algorithm^26^ to quantitatively evaluate the diets of the individuals examined at Zorita de los Canes Castle. This package is primarily designed for estimating dietary contributions from food sources using measurements of δ^13^C and δ^15^N stable isotope ratios. In our study, we used it to estimate the proportional contributions of a mixture from known sources. The methodology utilized is elaborated in the works of Parnell et al.^62,63^. We considered six primary food groups: C_3_ crops (*Triticum aestivum-durum* or wheat, and *Hordeum vulgare L.* or barley), C_4_ crops (*Panicum miliaceum* or broomcorn millet), cattle (*Bos taurus*), ovicaprid, pigs (*Sus scrofa*), poultry (*Gallus gallus*), and marine and freshwater resources (*Anguilla anguilla, Argyrosomus regius*, *Dicentrarchus labrax, Diplodus sargus, Delphinidae, Merlucciidae,* Mugilidae, *Scombridae, Sparus aurata*, and *Zeus faber*). Due to the absence of marine remains and C_3_ and/or C_4_ plant seeds at Castillo de Zorita, we used the δ^13^C and δ^15^N values available in the literature — C_3_ cereals by Knipper et al.^27^, C_4_ cereals by Nitsch et al.^28^, Alexander et al.^17^, López-Costas and Müldner^29^, and Mion et al.^30^; and freshwater fish by Mion et al.^30^. Consequently, it is essential to acknowledge the limitations of our Bayesian model. The accuracy of this analysis may be compromised as it cannot be conducted with C_3_ and C_4_ grains, along with marine or river fish remains from Zorita de los Canes. It's essential to consider geographic and temporal variations, as they significantly influence the carbon (δ^13^C) and nitrogen (δ^15^N) isotopic values. Full method description, R Script, and results are in Supplementary Material S1 and S2.

### Supplementary Information

Supplementary Information 1.

Supplementary Information 2.

## Data Availability

All data generated or analysed during this study are included in this published article, and its supplementary information files.
