# Supplementary material for: Unravelling social status in the first medieval military order of the Iberian Peninsula using isotope analysis
Source: Sci Rep. 2024 May 14;14:11074. doi: 10.1038/s41598-024-61792-y (PMC11094010; doi:10.1038/s41598-024-61792-y)
Supplement: Supplementary file 1 — Supplementary Information 1. [file 41598_2024_61792_MOESM1_ESM.docx]

**Supplementary Material for**

**Unravelling Social Status in the First Medieval Military Order of the Iberian Peninsula Using Isotope Analysis**

Patxi Pérez-Ramallo^1,2*^, Carme Rissech^3*^, Lluis Lloveras^4^, Mary Lucas^2,5^, Dionisio Urbina^6^, Catalina Urquijo^6^, Patrick Roberts^2,7^

^1^Department of Archaeology and Cultural History, NTNU University Museum, Erling Skakkes gt 47b, 7491, Trondheim, Norway

^2^isoTROPIC Research Group, Department of Archaeology, Max Planck Institute of Geoanthropology. Kahlaische Str. 10, 07745, Jena, Germany.

^3^Unitat d’Anatomia I Embriologia Humana, Dept de CiènciesMèdiques Bàsiques, Facultat de Medicina i Ciències de LaSalut, Universitat Rovira i Virgily, 43201, Reus, Tarragona, Spain.

^4^SERP, Departament ’'Història i Arqueologia, Universitat de Barcelona, 08001, Barcelona, Spain.

^5^Arctic University Museum of Norway, UiT-the Arctic University of Norway, 9006, Tromsø, Norway

^6^Archaeologists, Independent Researcher. ArchaeoSpain directors. Juan Gavala 2, 16555 Carrascosa del Campo Cuenca, Spain.

^7^Department of Archaeology, Max Planck Institute of Geoanthropology. Kahlaische Str. 10, 07745, Jena, Germany.

*Correspondence to: Patxi Pérez-Ramallo ([patxi.p.ramallo@ntnu.no](mailto:patxi.p.ramallo@ntnu.no)) and Carme Rissech ([carme.rissech@urv.cat](mailto:carme.rissech@urv.cat))

**This PDF file includes:**

**Supplementary Material S2 ‘Simmr’ R Bayesian Model Results…3**

**Tables:**

- Table S2.1. δ^13^C and δ^15^N values (Mean, SD, and number) of the food sources used in the Simmr R Bayesian Model for the human individuals of Zorita de los Canes…3
- Table S2.2. Results algorithm converged…3
- Table S2.3. Results posterior predicted check…4
- Table S2.4. Means, SD and credible intervals of food sources among individuals of Zorita de los Canes…6
- Table S2.5. Quartiles intervals of food sources among individuals of Zorita de los Canes…7

**Figures:**

- Figure S2.1. Posterior predicted check… 4
- Figure S2.2. Prior and posterior distributions food sources among individuals of

Zorita de los Canes… 5

- Figure S2.3. Density plot food sources among individuals of Zorita de los Canes…7
- Figure S2.4. Matrix plot of density food sources among individuals of Zorita de los Canes…8

**Supplementary Material S3. Statistical tests comparison -PAST software- (Hammer et al., 2001)…11**

**S3.1. Statistical comparison δ^15^N and δ^13^C values between terrestrial fauna (*Sus scrofa, Oryctolagus cuniculus, Bos taurus, Gallus gallus,* and ovicaprine)**

- Table S.3.1. Mann-Whitney pairwise δ^15^N values (Bonferroni corrected p values)…11
- Table S.3.2. Mann-Whitney pairwise δ^13^C values (Bonferroni corrected p values)…12

**S3.2. Statistical comparison δ^15^N and δ^13^C values between individuals from present study, Kingdom of Aragon social elite individuals from Pérez-Ramallo et al. (2022), and the members of the Castile royal family (Seville) from Jiménez-Brobeil et al. (2016)**

- S.3.2.1. Mann-Whitney pairwise δ^13^C values (Bonferroni corrected p values)…12

**References…13**

**S2. ‘Simmr’ R Bayesian Model Results**

**Table S2.1. δ^13^C and δ^15^N values (Mean, SD, and number) of the food sources as reference sample used in the Simmr R Bayesian Model for the human individuals of Zorita de los Canes**

| **Food Sources** | **n** | **δ^13^C** | **SD** | **δ^15^N** | **SD** | **Reference** |
| --- | --- | --- | --- | --- | --- | --- |
| C_3_ plants | 103 | -22.6 | 1.2 | 5.4 | 1.7 | Knipper et al., 2020 |
| C_4_ plants | 12 | -10.4 | 0.3 | 6.8 | 2.7 | Nitsch et al., 2017 |
| *Sus domesticus* | 2 | -20.1 | 0.6 | 6.9 | 1.1 | Present study |
| *Oryctolagus cuniculus* | 5 | -21.6 | 0.3 | 4.1 | 0.7 | Present study |
| *Bos taurus* | 3 | -18.6 | 2.3 | 6.5 | 0.9 | Present study |
| *Gallus gallus* | 3 | -19.9 | 0.1 | 7.5 | 0.5 | Present study |
| Ovicaprine | 6 | -20.6 | 0.4 | 6.0 | 1.0 | Present study |
| Marine Fish | 66 | -11.1 | 2.4 | 11.0 | 2.3 | Alexander et al., 2015; López-Costas & Müldner, 2016; and Mion et al., 2022 |
| Freshwater Fish | 42 | -14.1 | 5.3 | 12.5 | 2.2 | Mion et al., 2022 |

**Checking the algorithm converged**

Markov chain Monte Carlo (MCMC) works by repeatedly guessing the values of the dietary proportions and find those values which fit the data best.

As the values are close to 1, the model run Markov chain Monte Carlo had converged properly.

**Table S2.2. Results algorithm converged**

| Deviance | C_3_ plants | C_4_ plants | *Sus domesticus* |
| --- | --- | --- | --- |
| 1 | 1 | 1 | 1 |
| *Oryctolagus cuniculus* | *Bos taurus* | *Gallus Gallus* | Ovicaprine |
| 1 | 1 | 1 | 1 |
| Marine Fish | Freshwater Fish | SD [**δ^13^C**] | SD [**δ^15^N**] |
| 1 | 1 | 1 | 1 |

### Figure S2.1. Posterior predicted check


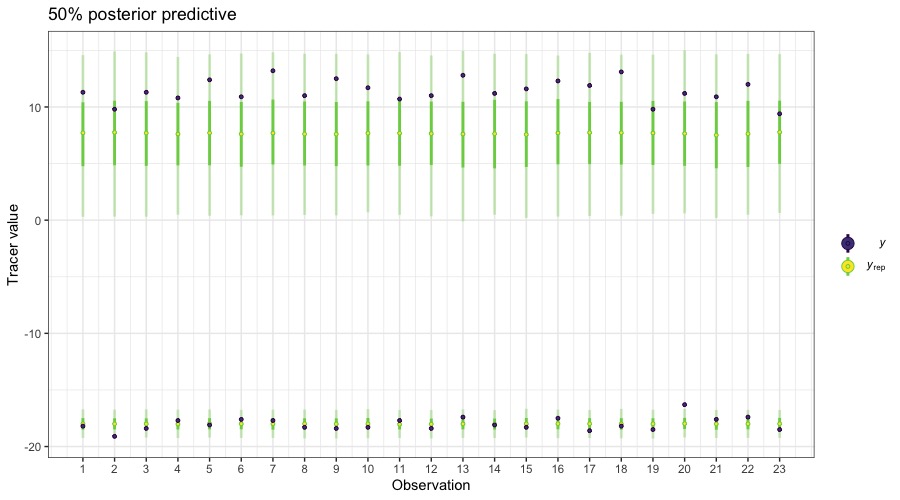


**Table S2.3. Results posterior predicted check**

Ref. Interval.1 Interval.2 Data Outside

1 -18.500044 -17.48718 -18.2 FALSE

2 -18.485194 -17.50407 -19.1 TRUE

3 -18.482556 -17.51322 -18.4 FALSE

4 -18.492927 -17.53092 -17.7 FALSE

5 -18.484839 -17.49494 -18.1 FALSE

6 -18.475643 -17.49389 -17.6 FALSE

7 -18.507894 -17.47914 -17.7 FALSE

8 -18.509633 -17.50918 -18.3 FALSE

9 -18.491941 -17.50696 -18.4 FALSE

10 -18.481958 -17.47496 -18.3 FALSE

11 -18.491900 -17.50079 -17.7 FALSE

12 -18.497395 -17.53134 -18.4 FALSE

13 -18.482228 -17.48756 -17.4 TRUE

14 -18.497417 -17.53045 -18.1 FALSE

15 -18.484546 -17.47732 -18.3 FALSE

16 -18.475562 -17.50017 -17.5 TRUE

17 -18.482735 -17.49108 -18.6 TRUE

18 -18.504877 -17.49293 -18.2 FALSE

19 -18.507828 -17.49968 -18.5 FALSE

20 -18.449160 -17.46758 -16.3 TRUE

21 -18.511714 -17.50518 -17.6 FALSE

22 -18.479042 -17.50383 -17.4 TRUE

23 -18.489020 -17.47291 -18.5 TRUE

24 4.771179 10.41323 11.3 TRUE

25 4.852296 10.55204 9.8 FALSE

26 4.796197 10.51848 11.3 TRUE

27 4.843996 10.36685 10.8 TRUE

28 4.851199 10.53607 12.4 TRUE

29 4.721166 10.44246 10.9 TRUE

30 4.917471 10.65233 13.2 TRUE

31 4.831069 10.48434 11.0 TRUE

32 4.773632 10.43950 12.5 TRUE

33 4.830670 10.49877 11.7 TRUE

34 4.810601 10.52440 10.7 TRUE

35 4.879133 10.47420 11.0 TRUE

36 4.654560 10.45295 12.8 TRUE

37 4.578012 10.63493 11.2 TRUE

38 4.699231 10.50057 11.6 TRUE

39 4.934029 10.69744 12.3 TRUE

40 4.951981 10.44997 11.9 TRUE

41 4.914241 10.45478 13.1 TRUE

42 4.879772 10.53314 9.8 FALSE

43 4.801746 10.48081 11.2 TRUE

44 4.584006 10.43639 10.9 TRUE

45 4.705466 10.54103 12.0 TRUE

46 4.973236 10.55619 9.4 FALSE

Prop_outside

[1] 0.5869565

**Exploring the results**

**Figure S2.2. Prior and posterior distributions food sources among individuals of Zorita de los Canes**


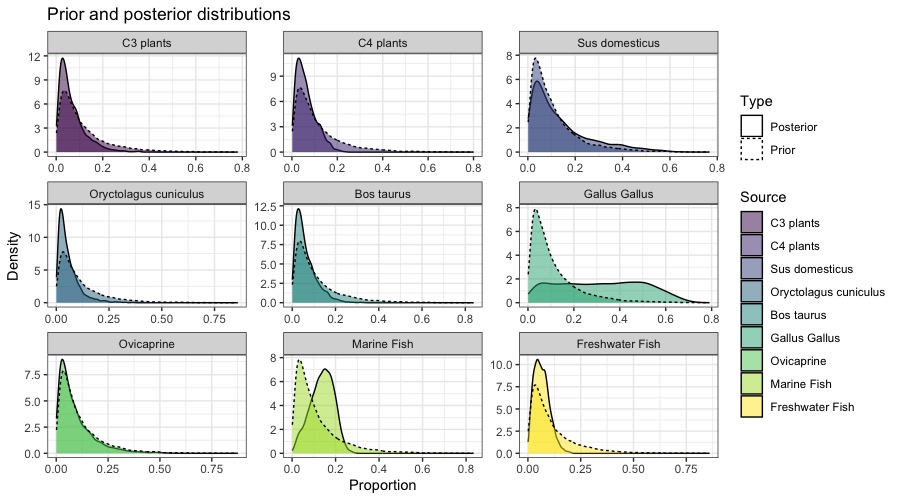


**Table S2.4. Means, SD and credible intervals of food sources** **among individuals of Zorita de los Canes**

|  | **Mean** | **SD** |
| --- | --- | --- |
| Deviance | 172.509 | 5.012 |
| C_3_ plants | 0.068 | 0.057 |
| C_4_ plants | 0.063 | 0.043 |
| *Sus domesticus* | 0.138 | 0.126 |
| *Oryctolagus cuniculus* | 0.057 | 0.050 |
| *Bos taurus* | 0.063 | 0.048 |
| *Gallus gallus* | 0.316 | 0.182 |
| Ovicaprine | 0.092 | 0.085 |
| Marine Fish | 0.138 | 0.053 |
| Freshwater Fish | 0.066 | 0.036 |
| SD[δ^13^C] | 0.441 | 0.168 |
| SD[δ^15^N] | 4.219 | 0.805 |

**Table S2.5. Quartiles intervals of food sources** **among individuals of Zorita de los Canes**

|  | 2.5% | 25% | 50% | 75% | 97.5% |
| --- | --- | --- | --- | --- | --- |
| Deviance | 163.910 | 168.956 | 171.989 | 176.145 | 183.606 |
| C_3_ plants | 0.008 | 0.028 | 0.051 | 0.092 | 0.217 |
| C_4_ plants | 0.008 | 0.029 | 0.053 | 0.087 | 0.166 |
| *Sus domesticus* | 0.010 | 0.043 | 0.096 | 0.191 | 0.473 |
| *Oryctolagus cuniculus* | 0.007 | 0.022 | 0.042 | 0.076 | 0.186 |
| *Bos taurus* | 0.007 | 0.027 | 0.049 | 0.086 | 0.187 |
| *Gallus gallus* | 0.024 | 0.159 | 0.318 | 0.470 | 0.637 |
| Ovicaprine | 0.008 | 0.032 | 0.064 | 0.125 | 0.327 |
| Marine Fish | 0.029 | 0.101 | 0.142 | 0.177 | 0.232 |
| Freshwater Fish | 0.011 | 0.038 | 0.062 | 0.089 | 0.145 |
| SD[δ^13^C] | 0.148 | 0.322 | 0.433 | 0.546 | 0.803 |
| SD[δ^15^N] | 2.982 | 3.722 | 4.206 | 4.748 | 6.141 |

**Figure S2.3. Posterior density plot food sources among individuals of Zorita de los Canes**


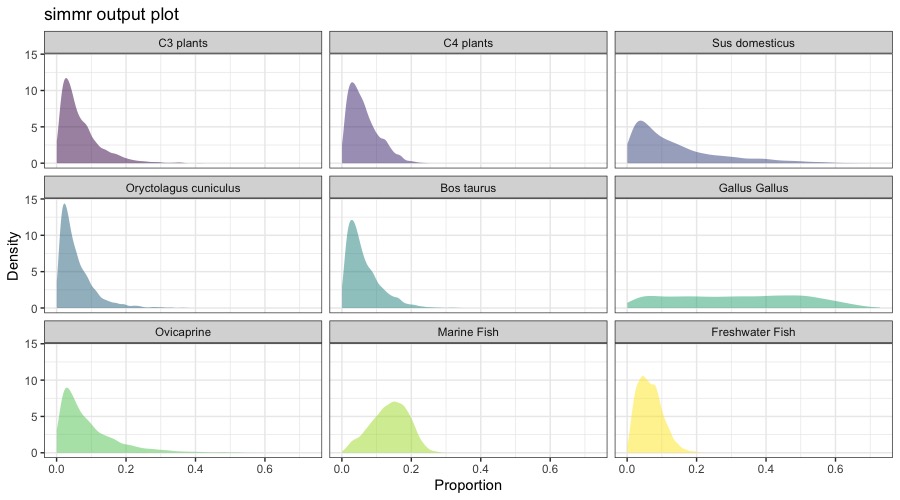


**Figure S2.4. Matrix plot of density food sources among individuals of Zorita de los Canes**


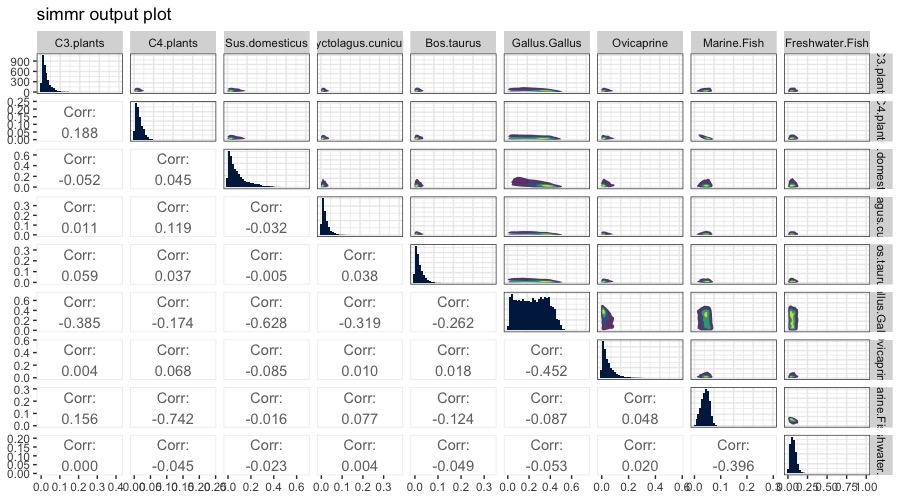


**Probability of most popular food sources among the individuals from Zorita de los Canes orderings are as follows:**

Gallus Gallus > Marine Fish > Ovicaprine > Freshwater Fish > C3 plants > Sus domesticus > Bos taurus > Oryctolagus cuniculus > C4 plants

0.0014

Gallus Gallus > Ovicaprine > Marine Fish > Freshwater Fish > C4 plants > Oryctolagus cuniculus > C3 plants > Sus domesticus > Bos taurus

0.0011

Gallus Gallus > Sus domesticus > Marine Fish > Freshwater Fish > Ovicaprine > Bos taurus > C3 plants > Oryctolagus cuniculus > C4 plants

0.0011

Gallus Gallus > Bos taurus > Marine Fish > Sus domesticus > C3 plants > Oryctolagus cuniculus > C4 plants > Freshwater Fish > Ovicaprine

0.0008

Gallus Gallus > Marine Fish > C3 plants > Freshwater Fish > Ovicaprine > C4 plants > Sus domesticus > Oryctolagus cuniculus > Bos taurus

0.0008

Gallus Gallus > Marine Fish > C3 plants > Sus domesticus > Freshwater Fish > Oryctolagus cuniculus > C4 plants > Bos taurus > Ovicaprine

0.0008

Gallus Gallus > Marine Fish > Freshwater Fish > Sus domesticus > Bos taurus > Oryctolagus cuniculus > Ovicaprine > C3 plants > C4 plants

0.0008

Gallus Gallus > Marine Fish > Oryctolagus cuniculus > Freshwater Fish > C4 plants > Ovicaprine > Bos taurus > C3 plants > Sus domesticus

0.0008

Gallus Gallus > Marine Fish > Ovicaprine > Oryctolagus cuniculus > Freshwater Fish > C3 plants > Sus domesticus > C4 plants > Bos taurus

0.0008

Gallus Gallus > Marine Fish > Ovicaprine > Oryctolagus cuniculus > Sus domesticus > Freshwater Fish > Bos taurus > C3 plants > C4 plants

0.0008

Gallus Gallus > Marine Fish > Ovicaprine > Oryctolagus cuniculus > Sus domesticus > Freshwater Fish > C3 plants > Bos taurus > C4 plants

0.0008

Gallus Gallus > Marine Fish > Sus domesticus > C3 plants > Freshwater Fish > Oryctolagus cuniculus > Bos taurus > Ovicaprine > C4 plants

0.0008

Gallus Gallus > Marine Fish > Sus domesticus > C3 plants > Freshwater Fish > Ovicaprine > Bos taurus > Oryctolagus cuniculus > C4 plants

0.0008

Gallus Gallus > Marine Fish > Sus domesticus > Freshwater Fish > Ovicaprine > Oryctolagus cuniculus > C4 plants > C3 plants > Bos taurus

0.0008

Gallus Gallus > Marine Fish > Sus domesticus > Oryctolagus cuniculus > Freshwater Fish > C3 plants > C4 plants > Ovicaprine > Bos taurus

0.0008

Gallus Gallus > Marine Fish > Sus domesticus > Ovicaprine > C3 plants > Freshwater Fish > Bos taurus > Oryctolagus cuniculus > C4 plants

0.0008

Sus domesticus > Marine Fish > Oryctolagus cuniculus > Gallus Gallus > Ovicaprine > Freshwater Fish > Bos taurus > C4 plants > C3 plants

0.0008

C3 plants > Marine Fish > Sus domesticus > Freshwater Fish > Ovicaprine > Gallus Gallus > C4 plants > Oryctolagus cuniculus > Bos taurus

0.0006

Gallus Gallus > Bos taurus > Freshwater Fish > Marine Fish > Ovicaprine > C3 plants > C4 plants > Sus domesticus > Oryctolagus cuniculus

0.0006

Gallus Gallus > Bos taurus > Marine Fish > Freshwater Fish > Sus domesticus > C4 plants > Ovicaprine > C3 plants > Oryctolagus cuniculus

0.0006

Gallus Gallus > Bos taurus > Marine Fish > Oryctolagus cuniculus > Sus domesticus > Ovicaprine > Freshwater Fish > C4 plants > C3 plants

0.0006

Gallus Gallus > Bos taurus > Marine Fish > Ovicaprine > C3 plants > Oryctolagus cuniculus > C4 plants > Freshwater Fish > Sus domesticus

0.0006

Gallus Gallus > Bos taurus > Marine Fish > Sus domesticus > C4 plants > C3 plants > Oryctolagus cuniculus > Freshwater Fish > Ovicaprine

0.0006

Gallus Gallus > Bos taurus > Marine Fish > Sus domesticus > Freshwater Fish > Oryctolagus cuniculus > Ovicaprine > C4 plants > C3 plants

0.0006

Gallus Gallus > Bos taurus > Marine Fish > Sus domesticus > Ovicaprine > Freshwater Fish > Oryctolagus cuniculus > C4 plants > C3 plants

0.0006

Gallus Gallus > Bos taurus > Sus domesticus > Marine Fish > Freshwater Fish > C4 plants > Ovicaprine > C3 plants > Oryctolagus cuniculus

0.0006

Gallus Gallus > Freshwater Fish > Marine Fish > C4 plants > Bos taurus > Oryctolagus cuniculus > Sus domesticus > Ovicaprine > C3 plants

0.0006

Gallus Gallus > Freshwater Fish > Sus domesticus > Marine Fish > C4 plants > Ovicaprine > Bos taurus > Oryctolagus cuniculus > C3 plants

0.0006

Gallus Gallus > Marine Fish > Bos taurus > C3 plants > Freshwater Fish > C4 plants > Sus domesticus > Ovicaprine > Oryctolagus cuniculus

0.0006

Gallus Gallus > Marine Fish > Bos taurus > C3 plants > Ovicaprine > C4 plants > Oryctolagus cuniculus > Freshwater Fish > Sus domesticus

0.0006

---------------------------------------------------------------------------------------------------------------------

**S3. Statistical tests comparison -PAST software-** (Hammer et al., 2001)

**S3.1. Statistical comparison δ^15^N and δ^13^C values between terrestrial fauna (*Sus domesticus, Oryctolagus cuniculus, Bos taurus, Gallus gallus,* and ovicaprine)**

**Kruskal-Wallis test for equal medians δ^15^N values:**

H (chi2): 12.89

Hc (tie corrected): 12.91

p (same): 0.01173

*There is a significant difference between sample medians.*

**Table S.3.1. Mann-Whitney pairwise for equal medians in δ^15^N values (p value):**

|  | ***Sus domesticus*** | ***Oryctolagus cuniculus*** | ***Bos taurus*** | ***Gallus gallus*** | **Ovicaprine** |
| --- | --- | --- | --- | --- | --- |
| ***Sus domesticus*** | - | 0.0814 | 0.5536 | 0.7728 | 0.4018 |
| ***Oryctolagus cuniculus*** | 0.0814 | - | 0.0369 | 0.0369 | 0,0080 |
| ***Bos taurus*** | 0.5536 | 0.0369 | - | 0.3827 | 0.3642 |
| ***Gallus gallus*** | 0.7728 | 0.0369 | 0.3827 | - | 0.0919 |
| **Ovicaprine** | 0.4018 | 0,0080 | 0.3642 | 0.0919 | - |

**Kruskal-Wallis test for equal medians δ^13^C values:**

H (chi2): 12.97

Hc (tie corrected): 13.14

p (same): 0.01063

*There is a significant difference between sample medians.*

**Table S.3.2. Mann-Whitney pairwise for equal medians in δ^13^C values (p values):**

|  | ***Sus domesticus*** | ***Oryctolagus cuniculus*** | ***Bos taurus*** | ***Gallus gallus*** | **Ovicaprine** |
| --- | --- | --- | --- | --- | --- |
| ***Sus domesticus*** | - | 0.0786 | 0.5536 | 1 | 0.3932 |
| ***Oryctolagus cuniculus*** | 0.0786 | - | 0.0358 | 0.0358 | 0.0169 |
| ***Bos taurus*** | 0.5536 | 0.0358 | - | 0.6625 | 0.1486 |
| ***Gallus gallus*** | 1 | 0.0358 | 0.6625 | - | 0.03725 |
| **Ovicaprine** | 0.3932 | 0.0169 | 0.1486 | 0.03725 | - |

**S3.2. Statistical comparison δ^15^N and δ^13^C values between individuals from present study, Kingdom of Aragon social elite individuals from Pérez-Ramallo et al. (2022), and the members of the Castile royal family (Seville) from Jiménez-Brobeil et al. (2016)**

**Kruskal-Wallis test for equal medians δ^15^N values:**

H (chi2): 4,248

Hc (tie corrected): 4,256

p (same): 0,1191

*There is no significant difference between sample medians.*

**Kruskal-Wallis test for equal medians δ^13^C values:**

Kruskal-Wallis test for equal medians

H (chi2): 10,14

Hc (tie corrected): 10,22

p (same): 0,006026

*There is a significant difference between sample medians.*

**S.3.2.1. Mann-Whitney pairwise for equal medians in δ^13^C values (p values):**

|  | ***Zorita de los Canes*** | ***Castile Royal Family Seville*** | ***Social Elite Kingdom of Aragon*** |
| --- | --- | --- | --- |
| ***Zorita de los Canes*** | - | 0.0451 | 0.0404 |
| ***Castile Royal Family Seville*** | 0.0451 | - | 0.5212 |
| ***Social Elite Kingdom of Aragon*** | 0.0404 | 0.5212 | - |

**References**

Alexander, M. M., Gerrard, C. M., Gutiérrez, A., & Millard, A. R. (2015). Diet, society, and economy in late medieval Spain: Stable isotope evidence from Muslims and Christians from Gandía, Valencia. *American Journal of Physical Anthropology*, *156*(2), 263–273. https://doi.org/10.1002/ajpa.22647

Hammer, Ø., Harper, D. A. T., & Ryan, P. D. (2001). Past: Paleontological statistics software package for education and data analysis. *Palaeontologia Electronica*, *4*(1), 9.

Knipper, C., Rihuete-Herrada, C., Voltas, J., Held, P., Lull, V., Micó, R., Risch, R., & Alt, K. W. (2020). Reconstructing Bronze Age diets and farming strategies at the early Bronze Age sites of La Bastida and Gatas (southeast Iberia) using stable isotope analysis. *PLOS ONE*, *15*(3), e0229398. https://doi.org/10.1371/journal.pone.0229398

López-Costas, O., & Müldner, G. (2016). Fringes of the empire: Diet and cultural change at the Roman to post-Roman transition in NW Iberia. *American Journal of Physical Anthropology*, *161*(1), 141–154. https://doi.org/doi:10.1002/ajpa.23016

Mion, L., André, T., Mailloux, A., Sternberg, M., Morales Muniz, A., Rosello-Izquierdo, E., Llorente Rodríguez, L., & Herrscher, E. (2022). Contribution to Mediterranean medieval dietary studies: Stable carbon and nitrogen isotope data of marine and catadromous fish from Provence (9th–14th CE). *Data in Brief*, *41*, 108016. https://doi.org/10.1016/j.dib.2022.108016

Nitsch, E., Andreou, S., Creuzieux, A., Gardeisen, A., Halstead, P., Isaakidou, V., Karathanou, A., Kotsachristou, D., Nikolaidou, D., Papanthimou, A., Petridou, C., Triantaphyllou, S., Valamoti, S. M., Vasileiadou, A., & Bogaard, A. (2017). A bottom-up view of food surplus: using stable carbon and nitrogen isotope analysis to investigate agricultural strategies and diet at Bronze Age Archontiko and Thessaloniki Toumba, northern Greece. *World Archaeology*, *49*(1), 105–137. https://doi.org/10.1080/00438243.2016.1271745
